# *In situ* synthesis of Ag nanoparticles decorated on Ag_2_NCN heterostructures for increased visible-light photocatalytic degradation

**DOI:** 10.1039/d6ra04624j

**Published:** 2026-08-11

**Authors:** Haidong Yu, Xuehui Luo, Lijuan Zhang, Hua Deng, Zian Meng, Xiaohe Sun, Ping Qu, Min Lu

**Affiliations:** a Langfang Natural Resources Comprehensive Survey Center, China Geological Survey Langfang 065000 China lumin_cgs@163.com; b Center for Geophysical Survey, China Geological Survey Langfang 065000 China quping_cgs@163.com

## Abstract

There is still a great demand for visible-light-driven photocatalysts for the efficient degradation of organic pollutants. Silver cyanamide (Ag_2_NCN) was reduced by NaBH_4_ in this work to prepare an Ag/Ag_2_NCN heterostructure, yielding uniformly dispersed metallic Ag nanoparticles (Ag NPs) on the surface of Ag_2_NCN plates. The Ag-1/Ag_2_NCN composite exhibited markedly enhanced visible-light photocatalytic activity towards rhodamine B (Rhb), phenol, and tetracycline (TC), with a rate constant of degradation 6.6 times larger than that of pristine Ag_2_NCN for TC. Structural and optoelectronic characterization revealed that the surface plasmon resonance of Ag broadens light absorption, promotes photothermal conversion, and facilitates charge separation *via* a built-in electric field at the heterointerface. The trapping experiments proved that superoxide radicals and photo-generated holes are the dominant reactive species. The synergy between Ag and Ag_2_NCN provides a rational design strategy for advanced carbodiimide-based photocatalysts.

## Abstract 2

*In situ* reduced Ag/Ag_2_NCN heterostructure degrades TC 6.6 times faster than pristine Ag_2_NCN under visible light, via LSPR-enhanced absorption, charge separation, and photothermal effect.

## Graphical abstract

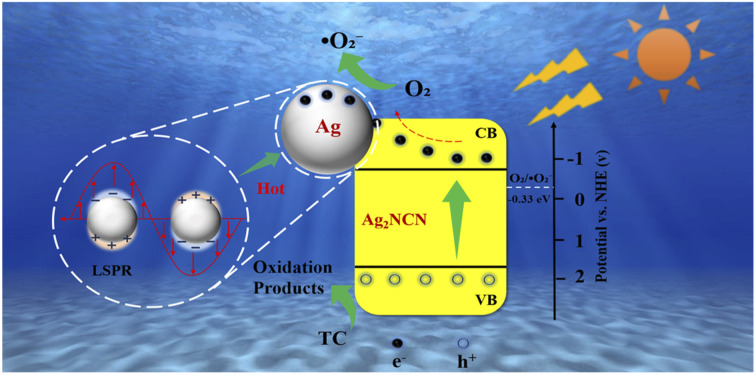

## 1. Introduction

The increased discharge of organic pollutants, including antibiotics, dyes and phenolic compounds into water bodies is a serious threat to ecological balance and public health.^1–6^ Semiconductor photocatalysis has emerged as a promising strategy to address this challenge, as it can harness solar energy to drive the degradation of these hazardous substances.^7–12^ Among various photocatalysts, silver-based compounds have drawn particular interest due to their favourable band structures and visible-light responsiveness.^13,14^ In recent years, silver carbodiimide (Ag_2_NCN), a compound featuring the unique [NCN]^2−^ anionic unit, has been identified as a potential candidate for visible-light photocatalysis.^15–17^ Nevertheless, the quick recombination of photo-generated electron–hole pairs, leading to a poor catalytic efficiency, continues to restrict its practical application.^16,18^

Building heterojunctions by depositing noble metal nanoparticles on a semiconductor host is an effective method for suppressing charge carrier recombination.^19–22^ Silver nanoparticles, in particular, are attractive for such modification because of their surface plasmon resonance (SPR) effect, which can prolong visible light absorption and facilitate charge separation.^23–26^ In this context, the formation of an Ag/Ag_2_NCN heterostructure is expected to combine the merits of both components: the active Ag_2_NCN matrix provides a suitable band structure for light absorption, while the deposited Ag clusters act as electron sinks and plasmonic photosensitisers. Nevertheless, the controlled synthesis of well-defined Ag/Ag_2_NCN interfaces remains challenging, and the detailed mechanism of charge transfer across such heterojunctions is not yet fully understood.

Herein, we report a simple ligand-induced precipitation method for preparing Ag_2_NCN,^27^ followed by an *in situ* surface reduction step to deposit Ag NPs with controlled loading amounts. The structure, morphology, surface chemistry, and optical properties of the resulting Ag/Ag_2_NCN composites are systematically characterised. The photocatalytic performance is evaluated through the degradation of RhB, phenol, and tetracycline under visible-light irradiation, and the enhancement mechanism is investigated *via* photoelectrochemical measurements and radical trapping experiments. This work provides a straightforward route to fabricate plasmonic Ag/Ag_2_NCN heterojunctions and offers insights into the role of Ag clusters in promoting charge separation and photothermal conversion.

## 2. Experimental

### 2.1. Materials

Silver nitrate (AgNO_3_), NaBH_4_, and cyanamide (H_2_NCN) were analytical reagent (A.R.) grade and purchased from Shanghai Aladdin Reagent Ltd. All chemicals were employed without further purification.

### 2.2. Synthesis of Ag_2_NCN *via* ligand-induced precipitation

In a typical synthesis, a precursor solution of [Ag(NH_3_)_2_]^+^ was first prepared. The precipitation of Ag_2_NCN was then induced by the dropwise addition of an aqueous solution of cyanamide (H_2_NCN) as the precipitating ligand. A quantity of 0.85 g of AgNO_3_ was weighed and dissolved in 20 mL of deionized water. Under continuous magnetic stirring, 125 mL of a 1.5 mol L^−1^ ammonia solution was promptly added, resulting in the formation of a homogeneous colorless solution. Subsequently, 5 mL of a 1 mol L^−1^ aqueous solution of H_2_NCN was introduced into the mixture, followed by stirring for 30 minutes, which led to the formation of a yellow precipitate. The precipitate was recovered by centrifugation, washed 3 times with deionized water and dried under vacuum at 60 °C for 12 hours.

### 2.3. Synthesis of Ag/Ag_2_NCN *via in situ* surface reduction

The as-synthesized Ag_2_NCN particles were dispersed in 20 mL of deionized water and ultrasonicated to form a homogeneous yellow suspension. Subsequently, a specified amount of NaBH_4_ was introduced into the suspension under continuous magnetic stirring for 0.5 hours, which reduced part of the Ag_2_NCN and yielded a brown precipitate. Centrifugation was used to collect the product, which was then cleaned three times with deionized water and vacuum-dried for 12 hours at 60 °C. Based on the amount of NaBH_4_ added, the theoretical deposition of Ag^0^ was calculated to be 0.5, 1, and 2 mol%. The resulting photocatalysts were accordingly named as Ag-*x*/Ag_2_NCN, where *x* corresponds to the theoretical Ag molar percentage (*x* = 0.5, 1, 2).

### 2.4. Characterization

The crystal structure of the as-synthesised samples was analysed by X-ray diffraction (XRD) on a Bruker instrument operated at 40 kV and 30 mA using Cu Kα radiation. Morphological characterisation was conducted using scanning electron microscopy (SEM) and transmission electron microscopy (TEM). Surface elemental composition and chemical states were investigated by X-ray photoelectron spectroscopy (XPS), with particular attention to Ag, C and N species. Optical properties were studied by UV-visible diffuse reflectance spectroscopy (UV-vis DRS) using an Agilent Cary 5000 spectrometer equipped with an integrating sphere; reflectance (*R*) data were used to evaluate pollutant degradation efficiency. Photoluminescence (PL) spectra were recorded on an Edinburgh Instruments FS5 spectrofluorometer, while surface photovoltage (SPV) measurements were employed to assess charge separation behaviour. Molecular structure and functional groups were identified using Fourier-transform infrared (FT-IR) and Raman spectroscopy. Electrochemical characterisation, such as transient photocurrent response, EIS, M–S analysis and CV, was conducted using a Chenhua electrochemical workstation.

### 2.5. Photocatalytic experiments

The photocatalytic activities of the as-prepared samples were evaluated by monitoring the degradation of typical pollutants, including tetracycline (TC), rhodamine B (RhB) and phenol. The TC, RhB, and phenol stock solution (30, 5, and 5 mg L^−1^) was prepared in deionized water without the addition of any buffer. The initial natural pH of the as-prepared solution, measured without pH adjustment, was found to be 6.8, and this solution was directly used for the subsequent kinetic experiments. In a typical procedure, the photocatalyst was distributed in the pollutant solution and magnetically stirred in the dark for 30 minutes to achieve adsorption–desorption equilibrium. The mixture was then irradiated under visible light (420–800 nm) using a 300 W xenon lamp. At given time intervals, 5 mL aliquots of the suspension were collected, filtered through a 0.22 µm membrane, and analysed by UV-vis spectroscopy to determine the residual pollutant concentration. The stability and reusability of Ag/Ag_2_NCN composites were evaluated by cycling testing.

## 3. Results and discussion

### 3.1. Physicochemical properties of Ag/Ag_2_NCN composites

**Fig. 1:**
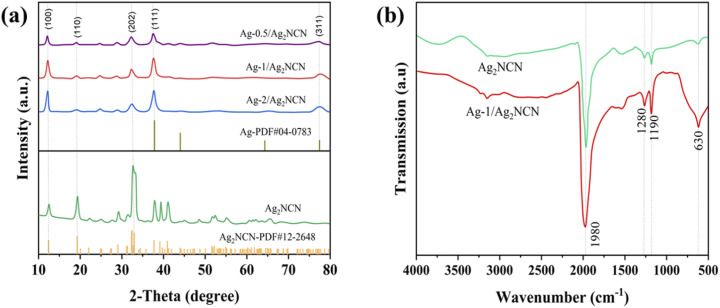
(a) X-ray diffraction patterns of Ag_2_NCN and Ag/Ag_2_NCN composites at varying mass ratios, (b) FT-IR spectra of Ag_2_NCN and Ag-1/Ag_2_NCN nanocomposites.

X-ray diffraction (XRD) was used to characterize the crystal structures of pristine Ag_2_NCN and the as-prepared Ag/Ag_2_NCN composites. As shown in Fig. 1a, the (100), (110) and (202) diffraction peaks of all samples agreed well with the standard pattern of Ag_2_NCN (JCPDS no. 12-2648), confirming the successful synthesis of the Ag_2_NCN matrix.^27^ The XRD pattern of the Ag/Ag_2_NCN composite is dominated by the characteristic peaks of Ag_2_NCN, indicating its role as the primary phase. Notably, two additional diffraction peaks are observed at 38.1° and 77.4°, which can be indexed to the (111) and (311) planes of metallic silver (JCPDS no. 04-0783).^28^ The result indicates that Ag NPs were reduced and deposited on the Ag_2_NCN surface.

To further verify the presence of the superatomic [NCN]^2−^ units, Fourier transform infrared (FT-IR) spectroscopy was performed (Fig. 1b). The characteristic absorption bands of the [NCN]^2−^ anion are evident in the spectra of both samples. Specifically, the peaks at 1980 cm^−1^ and 1280 cm^−1^ are assigned to the asymmetric and symmetric stretching vibrations, respectively, while the peaks at 1190 cm^−1^ and 630 cm^−1^ are attributed to the deformation vibrations of [NCN]^2−^.^29,30^ It is noteworthy that the intensity of the peaks at 630 cm^−1^ and 1190 cm^−1^ is significantly enhanced in the Ag-1/Ag_2_NCN composite. This enhancement is likely due to the interaction between the deposited Ag clusters and the Ag_2_NCN support. Crucially, the absence of new absorption bands or significant shifts suggests that no chemical bonds were formed between the metallic Ag and the Ag_2_NCN particles.

**Fig. 2:**
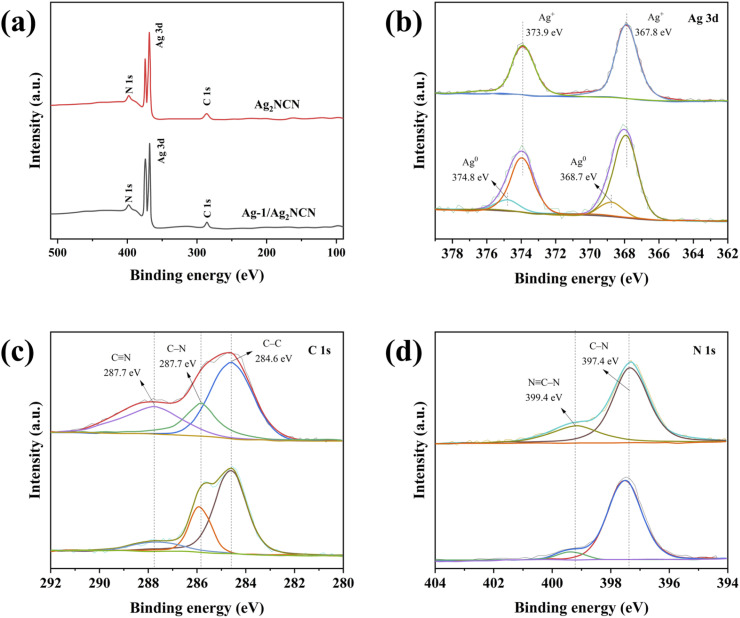
(a) XPS spectrum of Ag_2_NCN and Ag-1/Ag_2_NCN, XPS spectrum of (b) C 1s, (c) N 1s and (d) Ag 3d for Ag_2_NCN and Ag-1/Ag_2_NCN.

The elemental composition, chemical states and electronic environments of Ag_2_NCN and Ag-1/Ag_2_NCN were analysed by XPS. All binding energies were calibrated with reference to the adventitious C 1s peak at 284.6 eV to compensate for surface charging effects.^31^ As shown in Fig. 2a, the survey spectra confirm the presence of Ag, C, and N in both materials. High-resolution spectra of N 1s, C 1s, and Ag 3d are presented in Fig. 2b–d. The Ag 3d region exhibits two peaks at 367.9 and 373.8 eV, corresponding to Ag^+^ species. For Ag-1/Ag_2_NCN, two additional peaks appear at 368.8 and 374.8 eV, indicative of Ag^0^ nanoparticles, confirming the presence of metallic Ag NPs in the composite (Fig. 2b).^32^ The C 1s spectrum displays three contributions at 287.7, 285.7, and 284.6 eV, assigned to C≡N, C–N, and graphitic carbon, respectively (Fig. 2c).^33^ In the N 1s spectrum, the signal is deconvoluted into two components: a weaker peak at 399.4 eV, assigned to the nitrogen in the [N≡C–N] moiety (N≡) of Ag_2_NCN, and a dominant peak at 397.4 eV, corresponding to C–N bonded nitrogen (Fig. 2d).^34^ Notably, both the C 1s and N 1s peaks in Ag-1/Ag_2_NCN shift to higher binding energies relative to pristine Ag_2_NCN. This shift is consistent with electron transfer from Ag_2_NCN to Ag, where Ag_2_NCN is reduced and Ag gains electrons.^35^

**Fig. 3:**
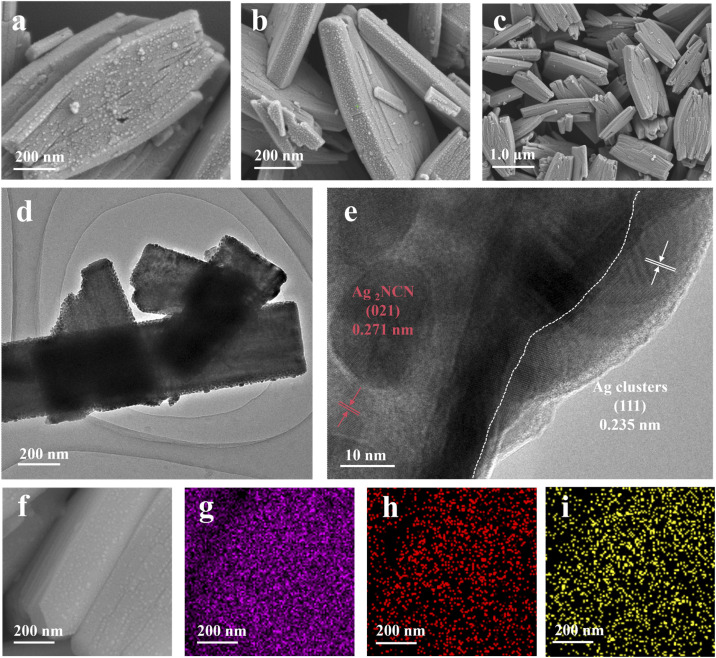
(a–c) SEM images, (d) TEM image, (e) HR-TEM image, and (f–i) elemental mapping of Ag-1/Ag_2_NCN.

The morphology of Ag-1/Ag_2_NCN was characterised by SEM and TEM, revealing that the Ag_2_NCN substrate was unaltered after Ag loading (Fig. 3a–d). The particles exhibited dimensions of approximately 2 µm in length and 0.2 µm in width, and the substrate's micrometer-scale solid architecture consisted of rectangular plates.^36,37^ In addition, discrete nanoparticles were evident in the SEM images, indicating the presence of Ag NPs. High resolution TEM (HRTEM) further revealed that the synthesized Ag NPs exhibited an oblong, coffee bean like shape with diameters between 10 and 20 nm.^38,39^ The measured lattice spacing of approximately 0.235 nm (Fig. 3e) is consistent with the (111) plane of metallic silver, confirming the presence of zero-valent Ag (0) nanostructures. Moreover, a distinct lattice fringe with an interplanar spacing of 0.271 nm corresponds to the (210) plane of Ag_2_NCN.^40^ Elemental mapping (Fig. 3f–i) provided a visual representation of the spatial distribution of Ag-1/Ag_2_NCN, with bright spots corresponding to Ag NPs dispersed uniformly across the material. These observations support the conclusion that coffee bean shaped Ag NPs were successfully grown on the Ag_2_NCN surface, consistent with initial design expectations. The Ag_2_NCN substrate appears to exert a pronounced size confinement effect during nanoparticle nucleation and growth, likely through *in situ* surface reduction that provides anchoring sites for nanoparticle formation.

The combined morphological, crystallographic, and elemental mapping results give compelling evidence for the nanoparticle character of Ag in the composite. These TEM discoveries, together with previous HRTEM, XRD, and XPS data, support the effective fabrication of the Ag/Ag_2_NCN heterostructure with well-defined interfaces.

### 3.2. Photocatalytic degradation performance

**Fig. 4:**
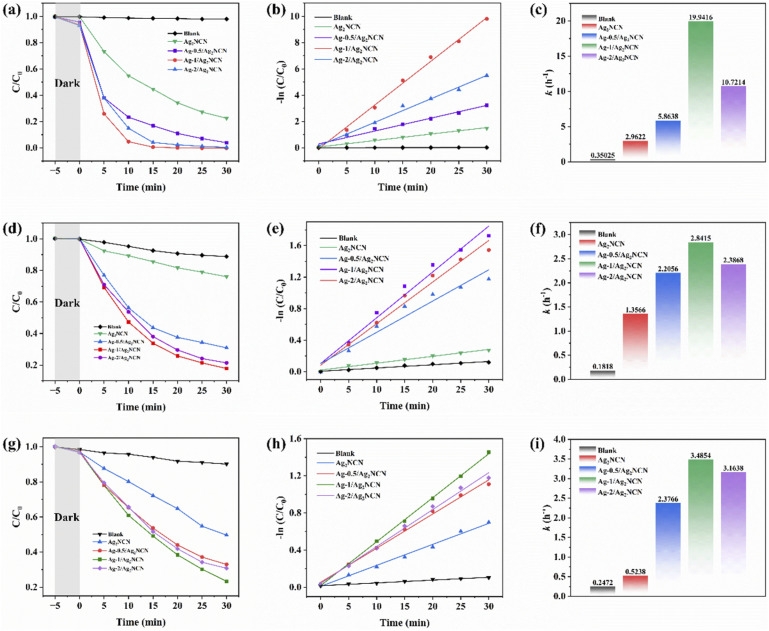
(a, d and g) Visible-light photocatalytic degradation of RhB, phenol and TC; (b, e and h) corresponding kinetic curves; (c, f and i) rate constants *k*.

The photocatalytic activity of the samples was evaluated under visible light (VL) irradiation by monitoring the degradation of RhB, phenol, and tetracycline (Fig. 4). As seen in Fig. 4a, with pristine Ag_2_NCN, only about 76% of RhB was degraded in 30 min. In contrast, the introduction of Ag NPs onto the Ag_2_NCN significantly enhanced the photocatalytic activity, particularly for the Ag-1/Ag_2_NCN heterojunction, which achieved complete RhB removal within 10 min. For quantitative comparison, the apparent rate constants (*k*) were derived from the pseudo-first-order kinetic model: −ln(*C*/*C*_0_) = *kt*, where *C*_0_ and *C* are the initial and time-dependent concentrations of RhB, respectively.^41^

As shown in Fig. 4b and c, the *k* values for Ag_2_NCN and Ag-1/Ag_2_NCN were determined to be 2.9622 and 19.9416 h^−1^, respectively, indicating that the degradation rate of Ag-1/Ag_2_NCN was approximately 6.6 times higher than that of pristine Ag_2_NCN. This enhancement is attributed to the efficient suppression of photogenerated carrier recombination in the Ag-1/Ag_2_NCN heterojunction, leading to improved photocatalytic degradation performance. In addition, the degradation performance was further evaluated using a colorless pollutant, phenol (5 mg L^−1^). As shown in Fig. 4d, approximately 80% of phenol was degraded within 30 min over the Ag-1/Ag_2_NCN heterojunction. Although the introduction of Ag clusters enhanced the catalytic activity by a factor of 2.2 (Fig. 4f), the overall degradation efficiency for phenol remained moderate. Subsequently, the photocatalytic degradation of TC (30 mg L^−1^) was also investigated. Compared to pristine Ag_2_NCN, the Ag/Ag_2_NCN heterojunction significantly improved the photocatalytic performance, with Ag-1/Ag_2_NCN exhibiting the highest enhancement—achieving a degradation efficiency approximately 6.6 times that of the pristine material. Among the three pollutants tested, TC was also degraded rapidly, suggesting that Ag_2_NCN-based materials are especially effective for colourless pollutants like TC. Fig. S6 shows the influence of solution pH on the photocatalytic degradation of TC over Ag-1/Ag_2_NCN under VL irradiation. Notably, the catalytic activity dropped significantly at extreme pH values, indicating that either acidic or basic conditions are unfavourable for the reaction. Nevertheless, the disappearance of the parent molecule does not signify its thorough mineralization, and the intermediate by-products could still be toxic to aquatic environments. As shown in Fig. S7, the TOC removal efficiencies were determined to be 51.3% for TC after 30 min of irradiation, 58.7% for RhB after 30 min, 41.5% for phenol after 30 min. The lower TOC removal compared to the UV-vis degradation efficiencies (TC: 75.5%, RhB: 96.2%, phenol: 73.8%) is attributable to the accumulation of refractory intermediates during the photocatalytic process, which requires longer irradiation time for complete mineralization.

**Fig. 5:**
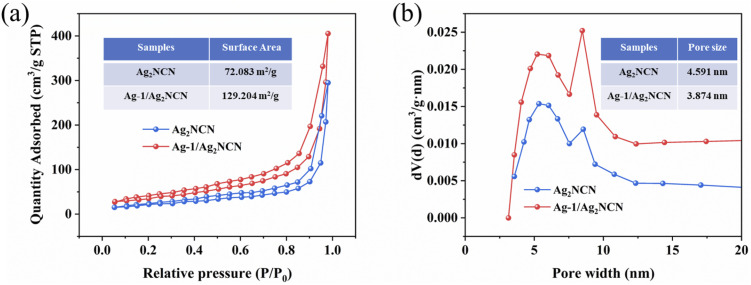
(a) N_2_ adsorption–desorption isotherms of Ag_2_NCN and Ag-1/Ag_2_NCN samples; (b) corresponding pore size distribution curves.

To better understand the enhanced photocatalytic performance of the Ag-1/Ag_2_NCN heterojunction, the specific surface area (SBET) and porous structure of Ag_2_NCN and Ag-1/Ag_2_NCN were characterized by nitrogen adsorption–desorption isotherms. As shown in Fig. 5a, both samples exhibit type IV isotherms, confirming the presence of the existence of both micropores and mesopores structures. The SBET values of Ag_2_NCN and Ag-1/Ag_2_NCN are 72.083 and 129.204 m^2^ g^−1^, respectively. As shown in Fig. 5b, the pore diameter decreases from 4.591 nm for pristine Ag_2_NCN to 3.874 nm for Ag-1/Ag_2_NCN, suggesting that the adsorption behavior is increasingly governed by micropores. The incorporation of Ag atoms appears to facilitate micropore formation, thereby providing additional active sites.^42,43^ The significant increase in both surface area and pore volume in Ag-1/Ag_2_NCN is likely due to the uniform dispersion of isolated Ag atoms, which effectively inhibits the agglomeration of Ag_2_NCN nanoparticles.^44^

The thermal stability of Ag_2_NCN and Ag/Ag_2_NCN nanocomposites was evaluated by thermogravimetric analysis (TGA) performed under air atmosphere from 30 to 800 °C at a heating rate of 10 °C min^−1^ (Fig. S5), with the aim of quantifying the silver content in the hybrid materials. A minor mass loss below 200 °C was observed, which is attributable to the desorption of surface-adsorbed water or residual volatile species. Thermal decomposition of bare Ag_2_NCN became pronounced at 529.8 °C and ceased at 764.1 °C. The same behaviour was observed for the Ag/Ag_2_NCN samples, indicating no substantial alteration in the degradation pathway. The residual mass fractions obtained from these profiles, as compiled in Table S1, were then employed to estimate the actual Ag loading within the Ag_2_NCN matrix.

### 3.3. Photoelectrochemical performance

**Fig. 6:**
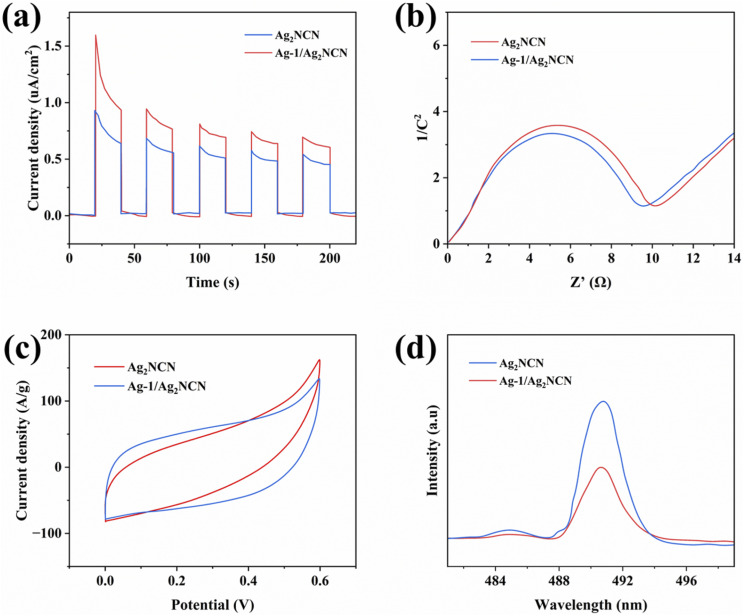
(a) Transient photocurrent responses, (b) EIS spectra, (c) cyclic voltammetry (CV) curves, and (d) steady-state photoluminescence (PL) spectra of the Ag_2_NCN and Ag-1/Ag_2_NCN heterojunction.

To investigate the dynamics of photogenerated charge carrier separation and transport, TPR and EIS measurements were conducted on Ag_2_NCN and the Ag-1/Ag_2_NCN heterojunction. As shown in Fig. 6a, the Ag-1/Ag_2_NCN electrode exhibits a markedly higher photocurrent density than pristine Ag_2_NCN over multiple on–off cycles, indicating more efficient separation of photogenerated carriers. Fig. 6b presents the EIS Nyquist plots; a smaller arc radius for the heterojunction reflects lower charge transfer resistance, suggesting that the incorporation of Ag facilitates interfacial charge migration and suppresses electron–hole recombination. Cyclic voltammetry (CV) curves in Fig. 6c further evaluate the photocatalytic activity. The larger integrated area of the CV curve for Ag-1/Ag_2_NCN compared to Ag_2_NCN demonstrates that the synergistic interaction between Ag and Ag_2_NCN enhances the redox capability during degradation. As shown in Fig. 6d, steady-state photoluminescence (PL) spectroscopy was used to probe the recombination of photogenerated electron–hole pairs. The lower emission intensity of Ag-1/Ag_2_NCN relative to pure Ag_2_NCN confirms that the introduction of Ag clusters effectively suppresses charge carrier recombination and promotes interfacial charge transfer, thereby boosting the photocatalytic performance.

**Fig. 7:**
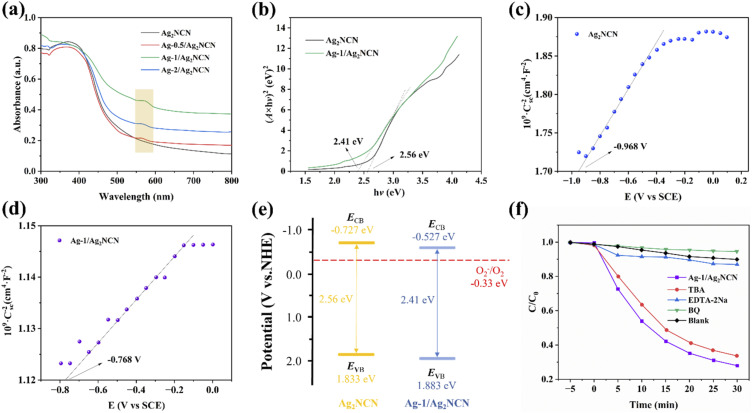
(a) UV-visible diffuse reflectance spectroscopy (UV-DRS) profiles of Ag_2_NCN and Ag/Ag_2_NCN nanoparticles; (b) corresponding Tauc plot analysis; (c and d) Mott–Schottky analysis of the two nanoparticle samples; (e) energy band alignment; (f) free radical trapping experiment on Ag-1/Ag_2_NCN.

UV-vis diffuse reflectance spectroscopy (UV-vis DRS) was employed to probe the optical properties and band gap energies (*E*_g_) of Ag_2_NCN and Ag-1/Ag_2_NCN. Notably, the Ag/Ag_2_NCN composites display a distinct absorption shoulder centred at approximately 550–600 nm (Fig. 7a), characteristic of the localized surface plasmon resonance (LSPR) arising from Ag NPs. In contrast to pristine Ag_2_NCN, the Ag/Ag_2_NCN hybrid shows significantly enhanced visible-light harvesting, along with a pronounced red shift in the absorption edge. These optical features are primarily attributable to the LSPR effect and the accompanying local electric field enhancement induced by the Ag NPs.^45,46^ Based on the Tauc plot analysis (Fig. 7b), the optical band gaps (*E*_g_) of Ag_2_NCN and Ag-1/Ag_2_NCN are determined to be 2.56 eV and 2.41 eV, respectively. The band gap (*E*_g_) was obtained from the Tauc relation: (*αh*ν)^1/*m*^ = *A*(*hν* − *E*_g_), where *α*, *h*, *ν* and *A* are the absorption coefficient, Planck's constant, light frequency and a proportionality constant, respectively.^47^ The exponent *m* equals 1/2 for a direct band gap and 2 for an indirect band gap. The Mott–Schottky analysis (Fig. 7c and d) reveals positive slopes for both samples, unequivocally confirming their n-type semiconductor nature. Consequently, an *m* value of 2 is adopted for the band gap determination, corresponding to indirect transition characteristics.^48^ The Mott–Schottky measurements further elucidate the band edge positions of the as-prepared photocatalysts. As presented in Fig. 7c and d, the flat band potentials, approximating the conduction band minima (ECB) for n-type semiconductors, are determined to be −0.968 V and −0.768 V *versus* saturated calomel electrode (SCE) for Ag_2_NCN and Ag-1/Ag_2_NCN, respectively. To reference these values against the normal hydrogen electrode (NHE) scale, a conversion factor of +0.241 V is applied (*E*(NHE) = *E*(SCE) + 0.241).^49^ Thus, the ECB values are calculated to be −0.727 V and −0.527 V *vs.* NHE for Ag_2_NCN and Ag-1/Ag_2_NCN, respectively. Employing the empirical relationship EVB = ECB + *E*_g_, the corresponding valence band potentials (EVB) are derived to be 1.833 eV and 1.883 eV *vs.* NHE, respectively.

To facilitate a comparative analysis, the energy band alignment of the as-prepared samples is schematically illustrated in Fig. 7e. Notably, the redox potential of O_2_/˙O_2_^−^ (−0.33 eV *vs.* NHE) falls within the band gap of the heterojunction, suggesting that superoxide radicals (˙O_2_^−^) may participate as active species in the pollutant degradation process.^50^ To elucidate the contribution of various reactive species, trapping experiments were conducted over the Ag-1/Ag_2_NCN heterojunction during tetracycline (TC) photodegradation, with results summarized in Fig. 7f and S1, *tert*-butyl alcohol (TBA), benzoquinone (BQ) and disodium ethylenediaminetetraacetate (EDTA-2Na) were used as scavengers for ˙OH, ˙O_2_^−^ and h^+^, respectively.^51^ The TC degradation rate was markedly suppressed upon the addition of BQ and EDTA-2Na, indicating that both ˙O_2_^−^ and h^+^ play dominant roles in the photocatalytic oxidation process. In contrast, the introduction of TBA resulted in only a slight inhibition of degradation activity, implying that ˙OH radicals contribute to a lesser extent. This observation can be rationalized by considering the energy level alignment: compared with the standard redox potential of H_2_O/˙OH (2.73 eV *vs.* NHE), the valence band potential of Ag-1/Ag_2_NCN is less positive, rendering ˙OH generation *via* hole oxidation thermodynamically unfavorable. The relative contributions of reactive species were quantitatively evaluated using radical scavenging experiments (Fig. S2). As summarised in Fig. S3, the contributions of ˙O_2_^−^, h^+^, and ˙OH to the overall degradation were determined to be 96%, 91%, and 11%, respectively. These results clearly indicate that superoxide radicals and photo-generated holes are the two dominant reactive species, while hydroxyl radicals play a negligible role. The Fermi level of Ag, by contrast, lies at a more negative potential than that of O_2_/˙O_2_^−^ (−0.33 eV *vs.* NHE), facilitating electron transfer to adsorbed O_2_ and thereby promoting ˙O_2_^−^ generation. This enhanced ˙O_2_^−^ production, in turn, underpins the superior photocatalytic activity of the heterojunction toward organic pollutant degradation.^52^

### 3.4. Analysis of the photocatalytic mechanism

**Fig. 8:**
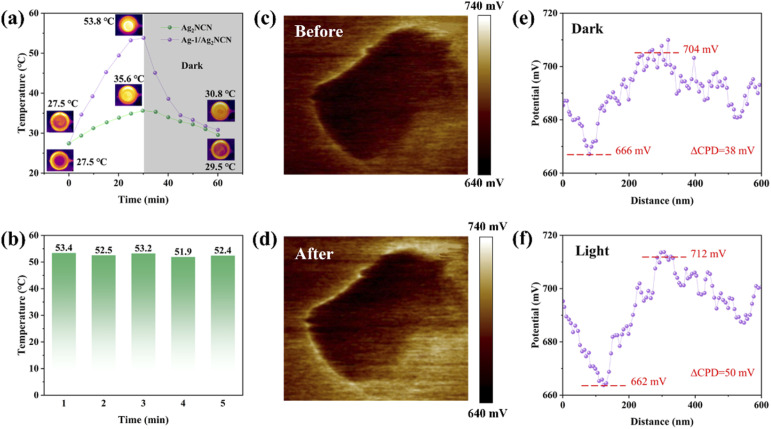
(a) Temperature change of Ag_2_NCN and Ag/Ag_2_NCN under/after VL (inset: IR images); (b) five heating/cooling cycles of Ag/Ag_2_NCN; (c and d) surface potential distribution before (c) and after (d) illumination; (e and f) surface potential curves in dark (e) and light (f).

As shown in Fig. 8a, the temperature evolution of the photocatalysts under visible light irradiation was tracked by thermal imaging. The Ag/Ag_2_NCN composite exhibited a final solution temperature approximately 20 °C higher than that of pristine Ag_2_NCN as irradiation proceeded. The temperature increase arises from the LSPR of Ag clusters, and five-cycle thermal cycling demonstrated the good thermal stability of the Ag_2_NCN heterostructure (Fig. 8b). The nearly identical thermal response in each cycle indicates excellent thermal stability of the composite material. To clarify whether the temperature rise induced by the LSPR effect contributes to the enhanced degradation, photocatalytic experiments were carried out at controlled temperatures of 25 °C and 50 °C using the Ag/Ag_2_NCN composite (Fig. S4). Notably, when the reaction temperature was increased from 25 °C to 50 °C, only a slight improvement of approximately 5% in the degradation efficiency was observed within 30 min. This marginal difference suggests that the photothermal effect plays a minor and non-essential role in the degradation process. Consequently, the substantial activity enhancement of Ag/Ag_2_NCN over pristine Ag_2_NCN is primarily attributed to the improved separation and transfer of photogenerated charge carriers, rather than to thermal activation. Kelvin probe force microscopy (KPFM) was used to verify the built-in electric field and elucidate the electron transfer dynamics of the Ag/Ag_2_NCN heterostructure. This technique measures the surface potential, allowing the direction of charge transfer to be determined (Fig. 8c and d). The surface potential of Ag/Ag_2_NCN changes significantly upon illumination (Fig. 8e and f). A ∼38 mV difference across the white-line interface in the dark suggests a built-in electric field that promotes photogenerated charge transfer. Illumination causes the positive potential of Ag/Ag_2_NCN to rise as photogenerated electrons migrate from Ag_2_NCN to Ag, while the heterojunction favours hole accumulation at the interface. The resulting ∼50 mV potential difference across the interface suggests a stronger built-in electric field and thus more efficient charge separation.

**Fig. 9:**
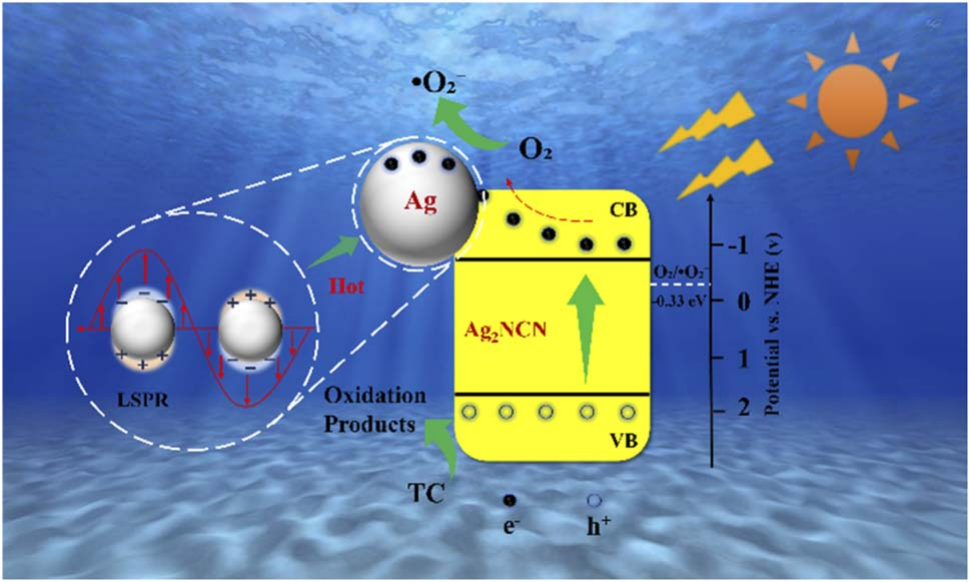
Schematic photocatalytic reaction mechanism of Ag/Ag_2_NCN nanocomposite.

A possible photocatalytic mechanism is proposed in Fig. 9. The LSPR of Ag NPs broadens the light absorption and enhances the absorption capability. Under visible light, electrons in the valence band of Ag_2_NCN are excited to the conduction band and then transfer to Ag across the heterojunction interface. The contact between Ag and Ag_2_NCN aligns their Fermi levels, inducing electron diffusion and positive charge accumulation at the interface, which results in upward band bending.^33^ Charge redistribution between Ag and Ag_2_NCN induces band edge bending and a built-in electric field, driving continuous electron migration from Ag_2_NCN to Ag. The electrons accepted by Ag effectively separate from holes. Meanwhile, the LSPR of Ag generates a photothermal effect that raises the temperature, further facilitating charge transfer and separation. The separated electrons react with surface-adsorbed O_2_ to form ˙O_2_^−^, while holes also serve as active species, together enabling more efficient TC degradation. In summary, the Ag/Ag_2_NCN heterostructure exhibits superior photothermal conversion and catalytic activity compared to pristine Ag_2_NCN.

## 4. Conclusions

In summary, the Ag/Ag_2_NCN heterostructure was successfully fabricated by *in situ* surface reduction of Ag_2_NCN with NaBH_4_. Metallic Ag clusters were uniformly dispersed on the Ag_2_NCN plates without altering the crystal structure or forming new chemical bonds. The Ag-1/Ag_2_NCN composite exhibited significantly enhanced visible-light photocatalytic activity for the degradation of RhB, phenol and TC, with a rate constant 6.6 times higher than that of pristine Ag_2_NCN for TC. The improved performance originates from the surface plasmon resonance of Ag, which broadens light absorption, promotes photothermal conversion, and facilitates charge separation *via* a built-in electric field at the heterointerface. ˙O_2_^−^ and h^+^ were identified as the dominant active species. The proposed mechanism highlights the synergistic effect between Ag and Ag_2_NCN, offering a strategy for designing efficient carbodiimide-based photocatalysts.

## Author contributions

Haidong Yu: conceptualization, methodology, investigation, writing – review and editing; Xuehui Luo: validation, investigation; Hua Deng: formal analysis, funding acquisition; Lijuan Zhang: validation, investigation; Xiaohe Sun: validation, investigation; Ping Qu: validation, investigation; Min Lu: conceptualization, supervision, funding acquisition.

## Conflicts of interest

There are no conflicts to declare.

## Supplementary Material

RA-OLF-D6RA04624J-s001

## Data Availability

The authors confirm that the data supporting the findings of this study are available within the article. Raw files supporting the findings of this study are available from the corresponding author on request. Supplementary information (SI) is available. See DOI: https://doi.org/10.1039/d6ra04624j.

## References

[cit1] Mondal S., Sahoo R., Das M. C. (2025). Small.

[cit2] Yahya S. N., Istiqomah N. I., Sari E. K., Asri N. S., Darmawan M. Y., Tumbelaka R. M., Adrianto N., Suharyadi E. (2025). Inorg. Chem. Commun..

[cit3] Nikpour S., Salehipour M., Rezaei S., Mogharabi-Manzari M. (2024). Biochem. Eng. J..

[cit4] Pan X., Ji J., Zhang N., Xing M. (2020). Chin. Chem. Lett..

[cit5] Zeng D., Yu C., Fan Q., Zeng J., Wei L., Li Z., Yang K., Ji H. (2020). Chem. Eng. J..

[cit6] Yu C., He H., Liu X., Zeng J., Liu Z. (2019). Chin. J. Catal..

[cit7] Apostolaki M.-A., Christoforou M.-K., Sakellis E., Tsipas P., Psycharis V., Gardelis S., Likodimos V. (2025). RSC Adv..

[cit8] Dong Y., Hu Y., Chen J. (2025). Sep. Purif. Technol..

[cit9] Xiao M., Zhang X., Liu X., Chen Z., Tai X., Wang X. (2025). ACS Macro Lett..

[cit10] Ren Y., Han S., Liu C., Feng Y., Li K., Song M. (2019). Int. J. Electrochem. Sci..

[cit11] Zeng D., Wang X., Yu Y., Qi M., Li S., Wang H., Cui Y., Tian F., Yang K., Yu C., Jiang Z., Guo Y. (2026). Appl. Catal., B.

[cit12] Meng X., Yu Z., Liu K., Tian J., Xie Y., Zeng D., Yu C. (2026). Chem. Eng. J..

[cit13] Jawhari A. H., Ismael A. M. (2025). RSC Adv..

[cit14] Zheng J., Deng Y., Chen W., Jiang X., Sun S., Deng C., Cai X., Cai J., Tan H. (2025). ACS Appl. Nano Mater..

[cit15] Xie Y., Zhuo Y., Liu S., Lin Y., Zuo D., Wu X., Li C., Wong P. K. (2020). Sol. RRL.

[cit16] Meng X., Yao P., Xu Y., Meng H., Zhang X. (2016). RSC Adv..

[cit17] Meng H., Li X., Zhang X., Liu Y., Xu Y., Han Y., Xu J. (2015). Dalton Trans..

[cit18] Zhao W., Pan J., Huang F. (2018). Chem. Commun..

[cit19] Yan H., Li W., Yang H., Yu Y., Lv C., Hou L., Zhang W., Lin D., Jiao S. (2024). Environ. Res..

[cit20] Xu D., Sun F., Liu F., Shao H., He W., Wang L., Ma Q., Yu W., Yu H., Dong X. (2024). J. Environ. Chem. Eng..

[cit21] Lai Y.-J., Lee D.-J. (2021). J. Taiwan Inst. Chem. Eng..

[cit22] Yu C., Chen F., Zeng D., Xie Y., Zhou W., Liu Z., Wei L., Yang K., Li D. (2019). Nanoscale.

[cit23] Jeffrin I A., Ramasamy P., Sathe V., Mahalingam U. (2025). J. Water Process Eng..

[cit24] Guo Y., Fu X., Xie Y., Zhu L., Liu R., Liu L. (2022). Opt. Mater..

[cit25] Iqbal T., Nafeesa N., Afsheen S., Yousaf M., Ahmad S., Bin sheeha B., Althomail O. W., Rab S. O., Qureshi M. T. (2025). Plasmonics.

[cit26] Zuo G., Ye H., Fang Y., Du J., Ding X. (2025). Catal. Lett..

[cit27] Yu H., Jiang H., Cao X., Yao S. (2023). RSC Adv..

[cit28] Wang Z., Wang D., Deng F., Liu X., Li X., Luo X., Peng Y., Zhang J., Zou J., Ding L., Zhang L. (2023). Chem. Eng. J..

[cit29] Fu Z., Wang Y., Li Z., Song T., Long B., Ali A., Deng G. J. (2021). J. Colloid Interface Sci..

[cit30] Meena P. L., Surela A. K., Chhachhia L. K., Kumar N. (2025). Ceram. Int..

[cit31] Silva-Quinones D., He C., Butera R. E., Wang G. T., Teplyakov A. V. (2020). Appl. Surf. Sci..

[cit32] Dong K., Shen C., Yan R., Liu Y., Zhuang C., Li S. (2024). Acta Phys.-Chim. Sin..

[cit33] Yu H., Cao X., Zhang S., Luo S., Feng L., An X., Jiang H., Yao S. (2023). Dalton Trans..

[cit34] Dong B., Li M., Chen S., Ding D., Wei W., Gao G., Ding S. (2017). ACS Appl. Mater. Interfaces.

[cit35] Yassin J. M., Taddesse A. M., Sánchez-Sánchez M. (2022). Appl. Surf. Sci..

[cit36] Yang Z., Wu B., Niu S., Zhai C., Xu T., Dang L., Qi X., Liu X., Shi R., Ma S., Yao M. (2024). Chem. Phys. Lett..

[cit37] Cao K., Ge X., Li S., Tian Z., Cui S., Guo G., Yang L., Li X., Wang Y., Bai S., Wei Q., Li W. (2025). RSC Adv..

[cit38] Fareid M. A., El-Sherbiny G. M., Askar A. A., Abdelaziz A. M., Hegazy A. M., Ab Aziz R., Hamada F. A. (2025). Biomolecules.

[cit39] Bai Y., Wang Y., Yang Y., Sun C., Wang H.-T., Lee C.-F., Li C.-Y., Zheng T., Jiang K., Han L. (2025). Chem. Eng. J..

[cit40] Kiani Z., Mirjalili S., Heydaryan K., Mohammadparast P., Aramjoo H., Bahraini F., Yousefinia A., Torabi M., Ghoreishi S. M., Fattahi M., Mortazavi-Derazkola S. (2024). J. Drug Delivery Sci. Technol..

[cit41] Chang Y., Guo J., Chen C., Tsay C. (2025). Ceram. Int..

[cit42] Li K., Yang X., Zhao T., Liu J., Liu J., Li Y., Li F., Tai X., Cao S. (2019). ChemistrySelect.

[cit43] Pan X., Kong F., Xing M. (2022). Res. Chem. Intermed..

[cit44] Wu F., Chen J., Hu J. (2022). J. Environ. Chem. Eng..

[cit45] Choudhary S., Mohapatra S. (2024). J. Photochem. Photobiol., A.

[cit46] Zhang L., Li Y., Li Q., Fan J., Carabineiro S. A. C., Lv K. (2021). Chem. Eng. J..

[cit47] Xu S., Zhang Y., Wang Y., Chen J., Zhou C., Mu Z., Zhang Z., Zhang J., Wang J., Zhang Q. (2023). J. Alloys Compd..

[cit48] Liu D., Jiang L., Chen D., Hao Z., Deng B., Sun Y., Liu X., Jia B., Chen L., Liu H. (2024). ACS Catal..

[cit49] Kawashima K., Márquez R. A., Son Y. J., Guo C., Vaidyula R. R., Smith L. A., Chukwuneke C. E., Mullins C. B. (2023). ACS Catal..

[cit50] Koppenol W. H., Stanbury D. M., Bounds P. L. (2010). Free Radical Biol. Med..

[cit51] Wang F., Zhao Q., Li H., Wu Q., Zhang L., Li Y., Qiao L., Yuan Y., Ma J., Wang P., Chen T. (2024). Chem. Eng. J..

[cit52] Chen Z., Huang L., Zhou X., Zhang Y., Chen X., Zhou C., Abdelsalam H., Chen W., Wang Z., Zhang Q. (2025). Environ. Res..

