# Supplementary material for: In situ synthesis of Ag nanoparticles decorated on Ag2NCN heterostructures for increased visible-light photocatalytic degradation
Source: RSC Adv. 2026 Aug 11;16(40):46603–12. doi: 10.1039/d6ra04624j (PMC13458688; doi:10.1039/d6ra04624j)
Supplement: Supplementary file 1 [file RA-016-D6RA04624J-s001.pdf]

Supplementary Information (SI) for RSC Advances.
This journal is © The Royal Society of Chemistry 2026

Supplementary Information for

In-situ synthesis of Ag nanoparticles decorated on Ag_2NCN heterostructures for increased visible-light photocatalytic degradation

Haidong Yu,[a] Xuehui Luo,[a] Lijuan Zhang,[a] Hua Deng,[a] Zian Meng,[a] Xiaohe Sun,[a] Ping Qu,*[b] and Min Lu,*[a]

a. *Langfang Natural Resources Comprehensive Survey Center, China Geological Survey, Langfang 065000, China.*

b. *Center for Geophysical Survey, China Geological Survey, Langfang 065000, China.*

*Correspondence E-mail: qupinq_cgs@163.com

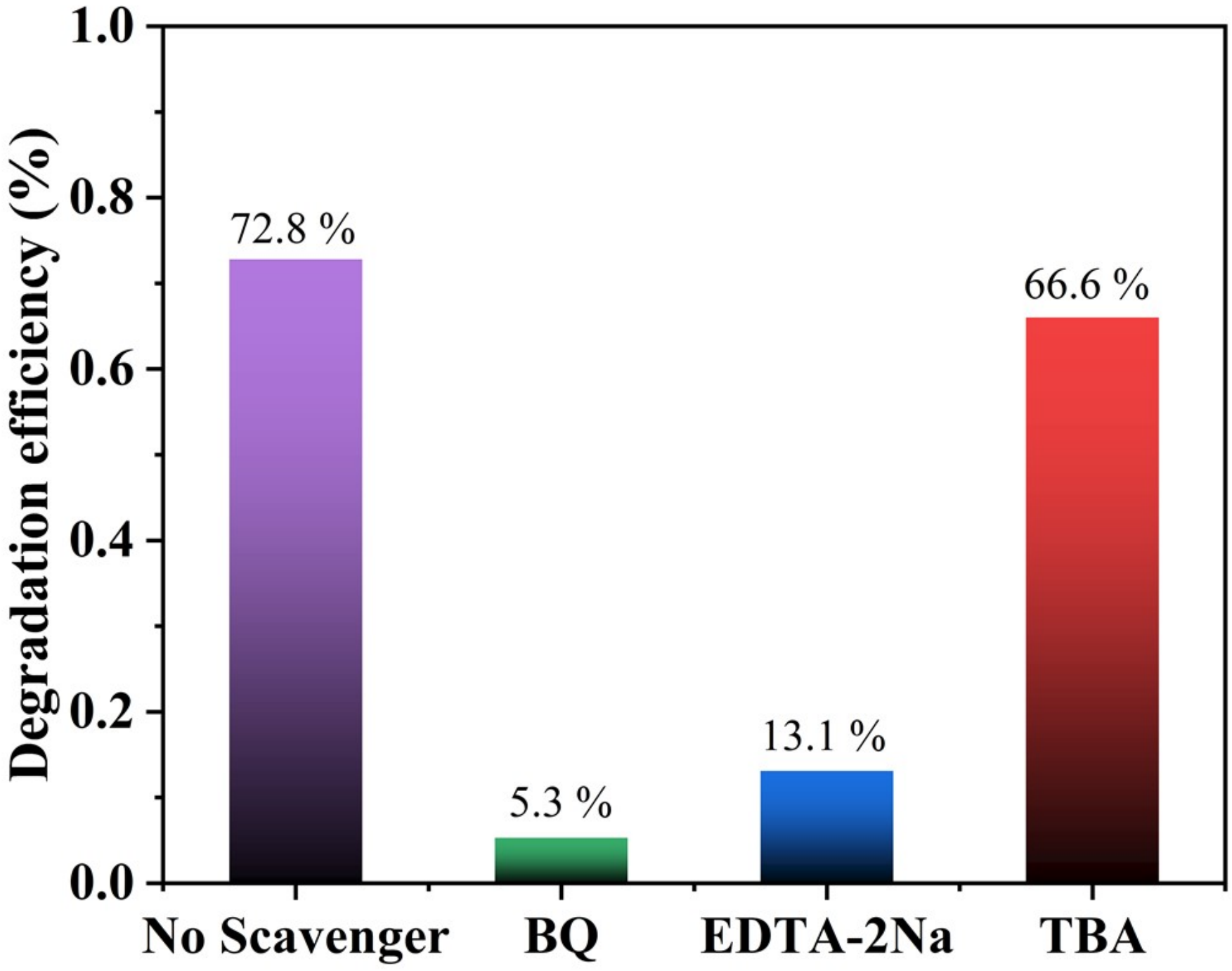

Fig. S1 The degradation efficiency of free radical trapping experiment on Ag-1/Ag_2NCN.

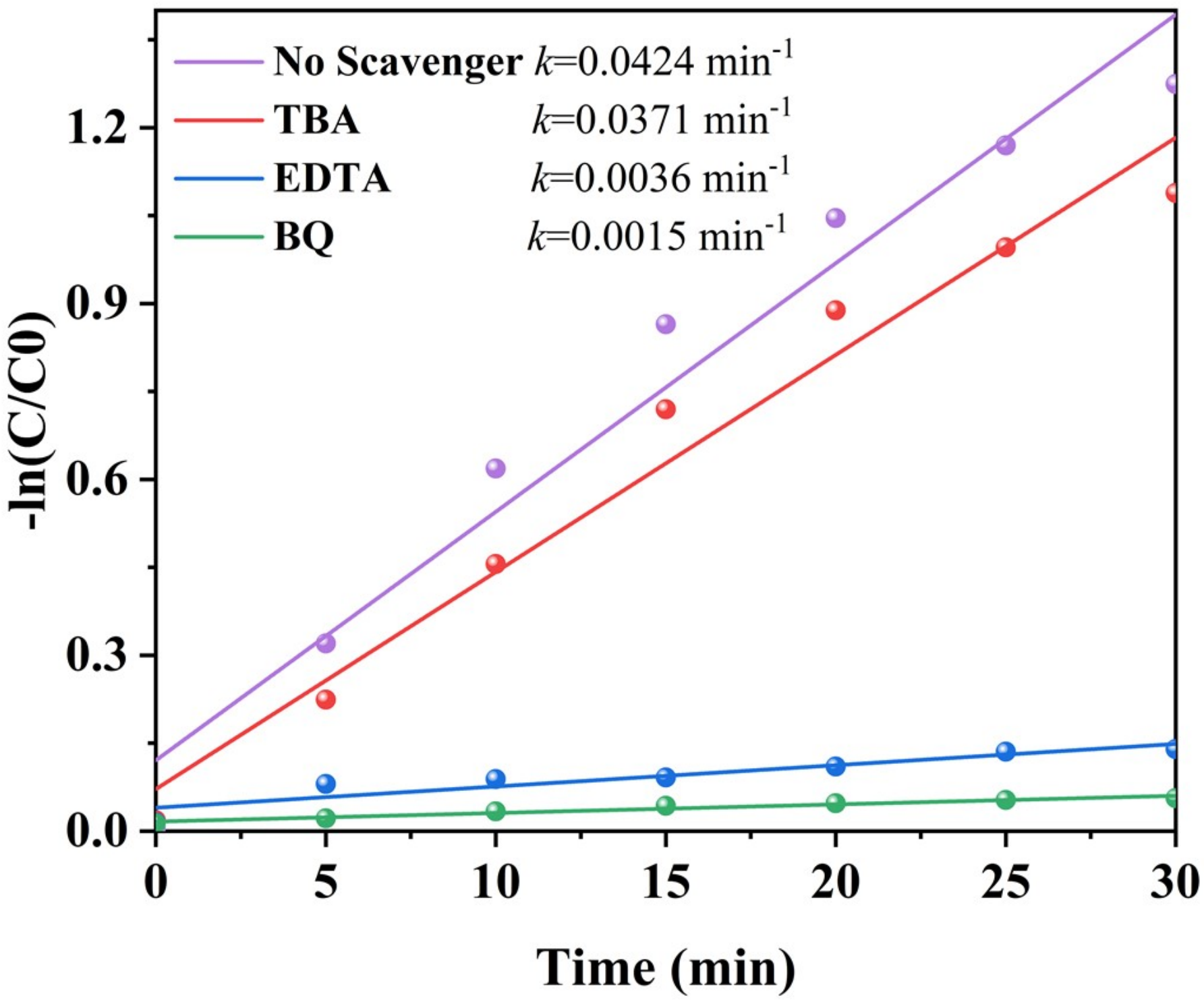

Fig. S2 The kinetic curves of free radical trapping experiment on Ag-1/Ag_2NCN.

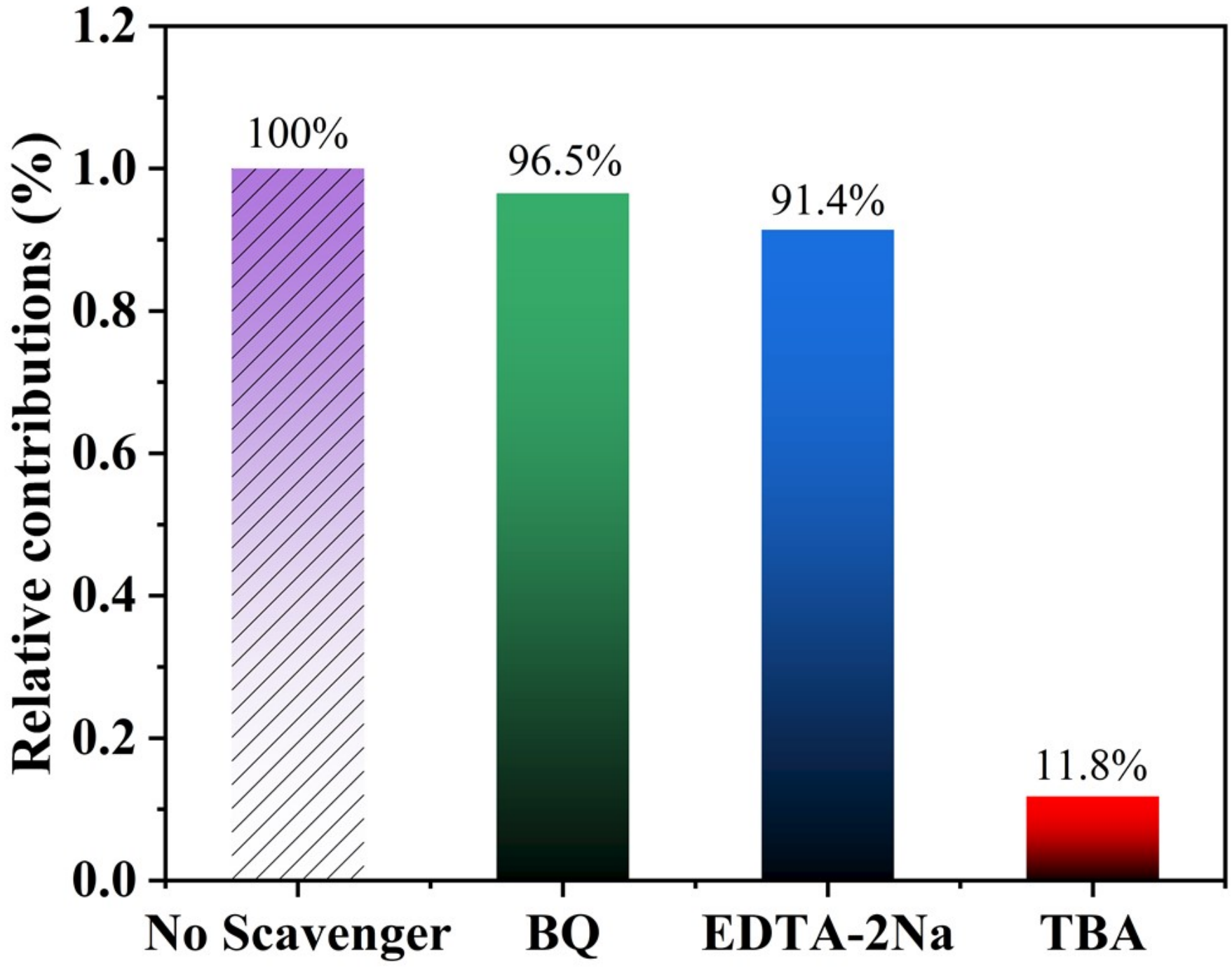

Fig. S3 The relative contributions of free radical trapping experiment.

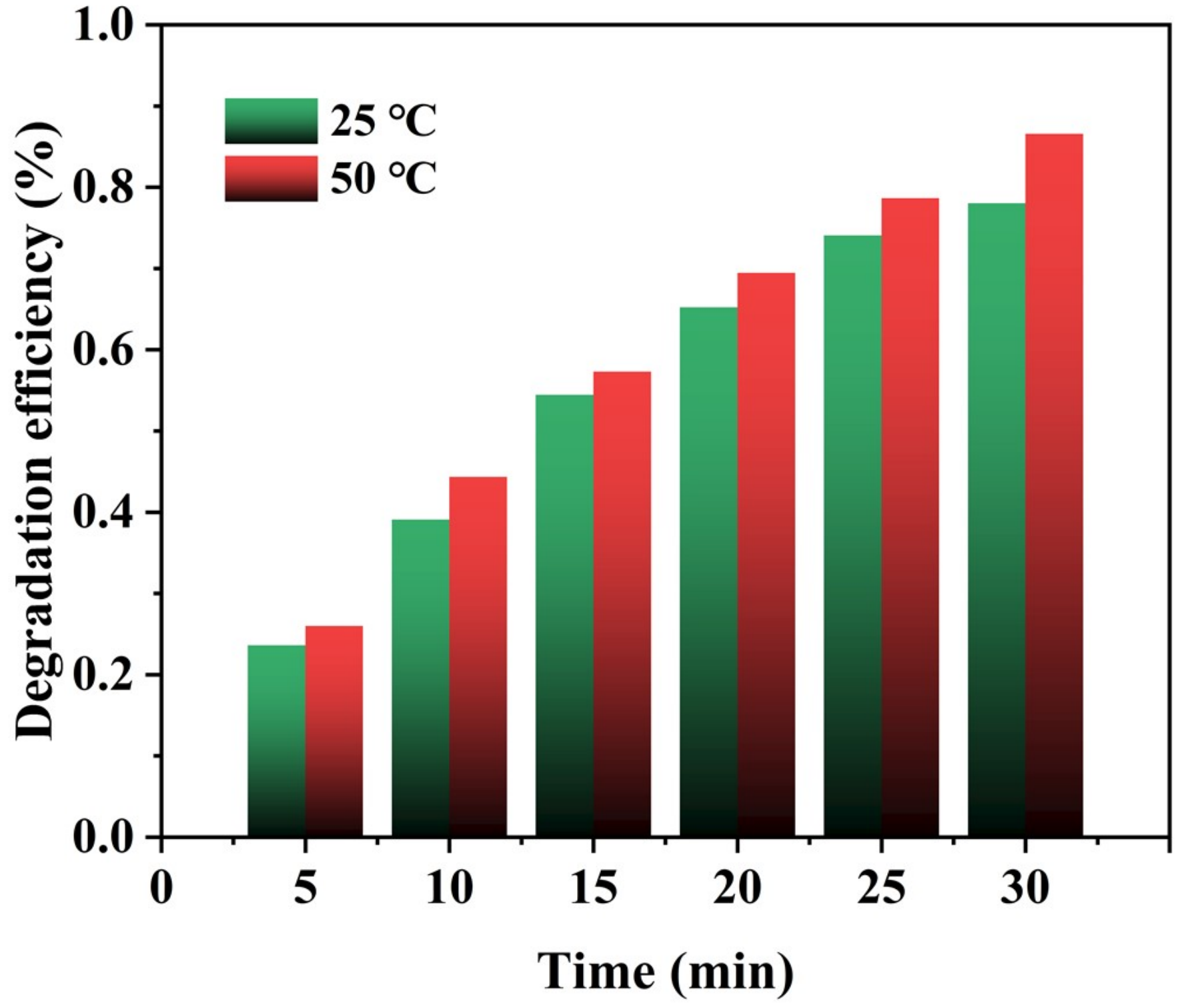

Fig. S4 Temperature-dependent TC photodegradation efficiency over Ag/Ag_2NCN.

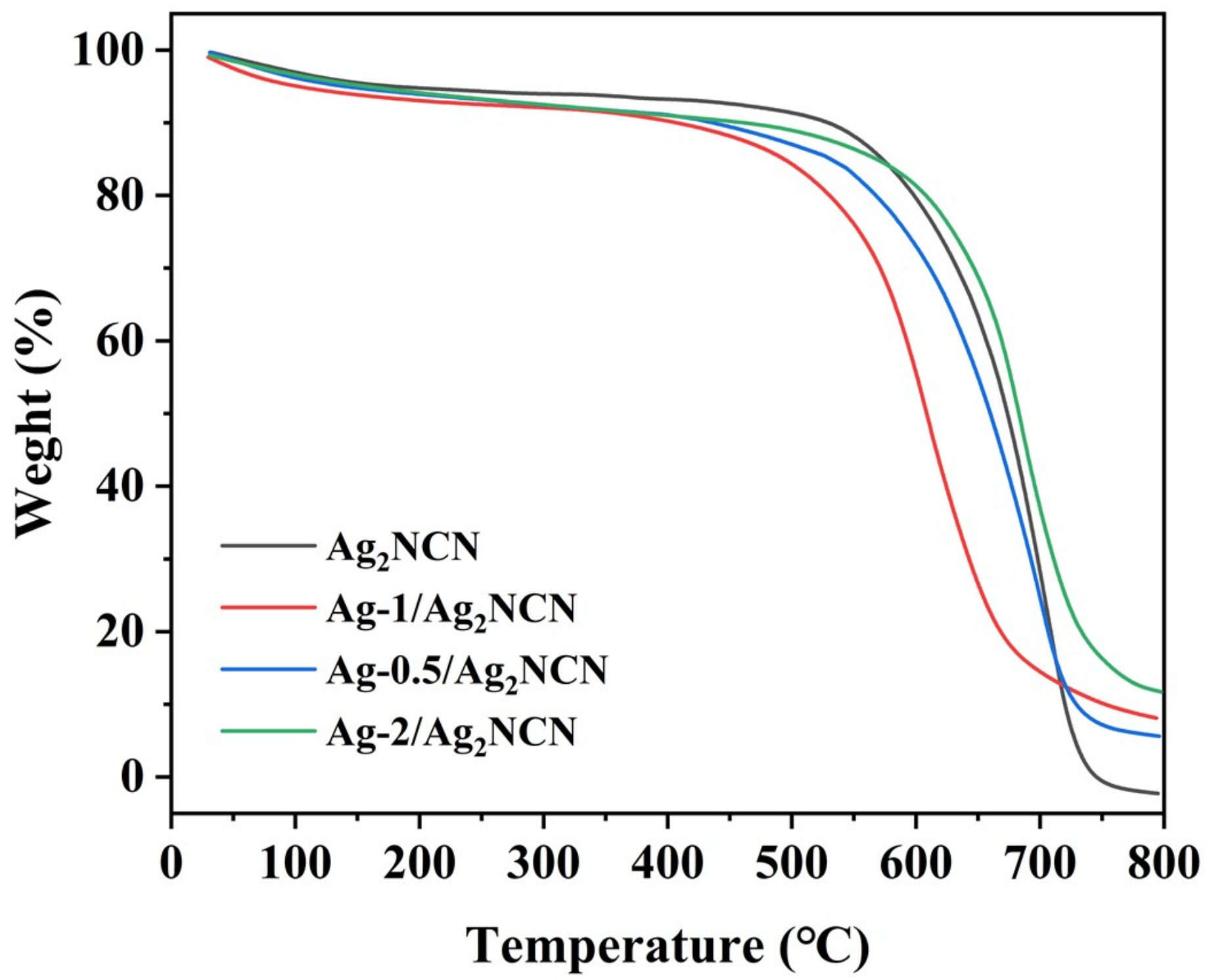

Fig. S5 TGA spectra of Ag and Ag/Ag_2NCN composites.

Table S1 Percentage of residual weight in TGA.

No.	Samples	Residual weight %
**1**	Ag_2NCN	0
**2**	Ag-0.5/Ag_2NCN	5.1
**3**	Ag-1/Ag_2NCN	8.9
**4**	Ag-2/Ag_2NCN	13.6

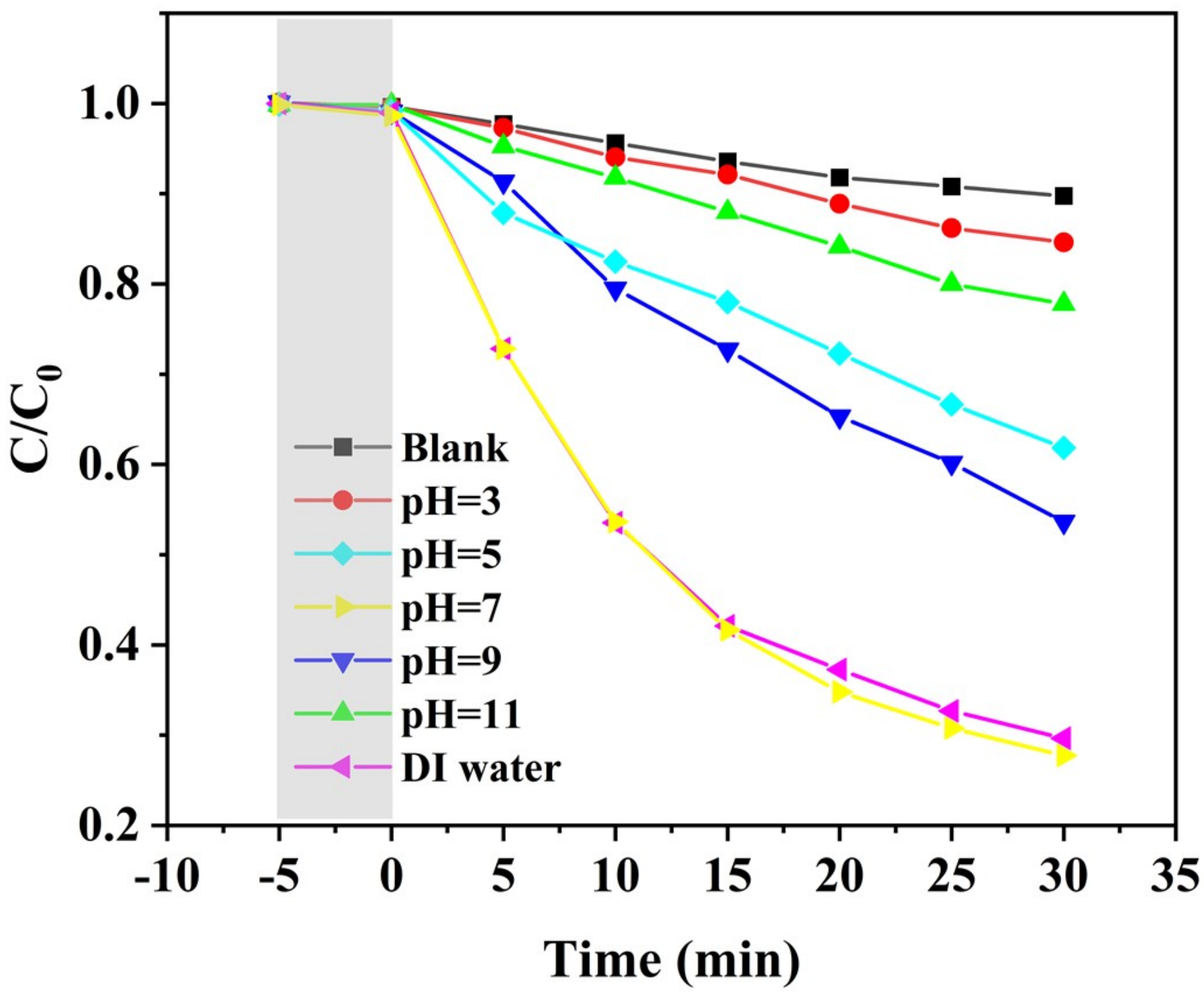

Fig. S6 Effects of pH under the VL irradiation for TC degradation.

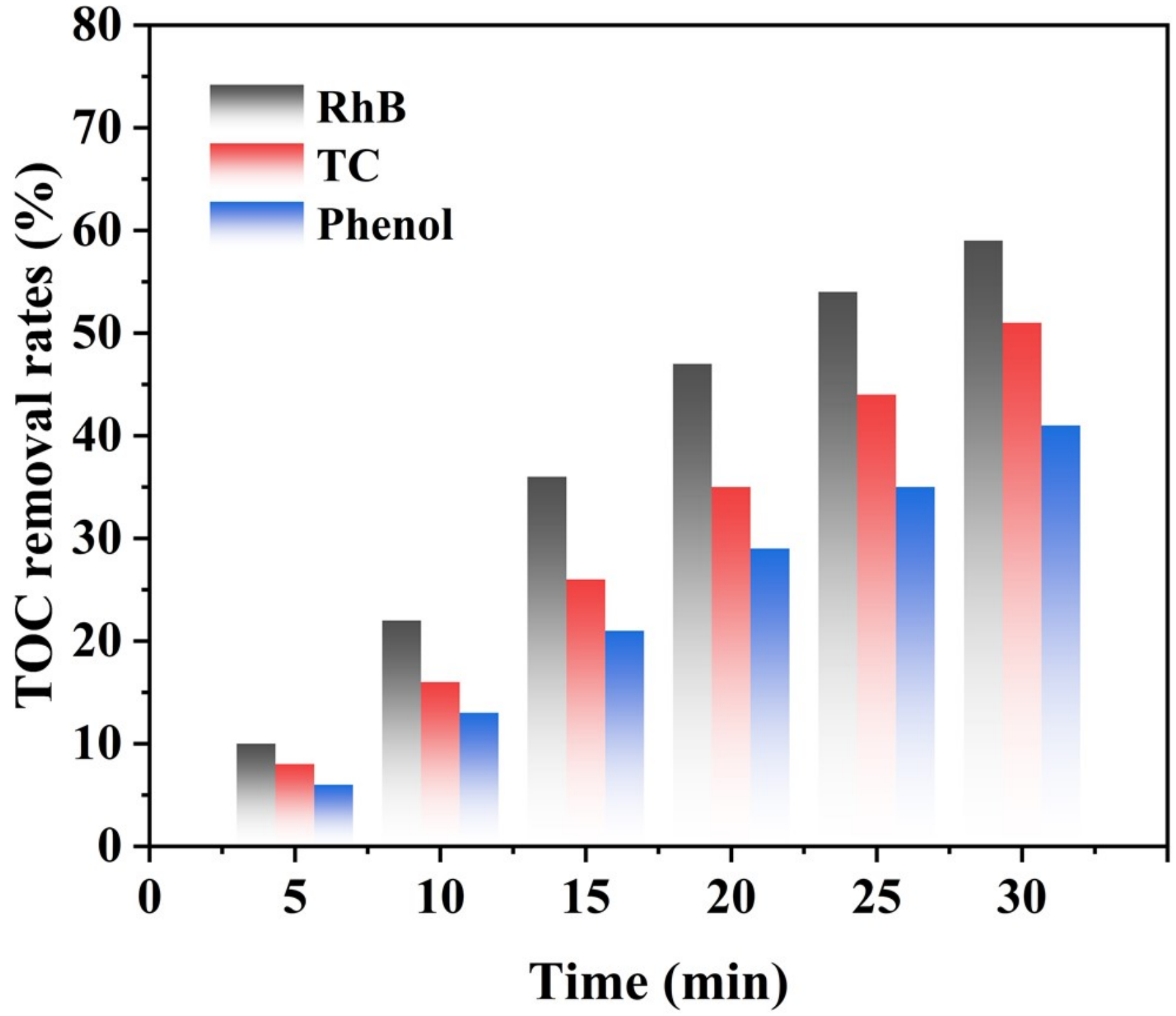

Fig. S7 TOC removal efficiencies of RhB, TC and phenol over Ag-1/Ag_2NCN.
